# Combining Electrochemical Scanning Tunneling Microscopy with Force Microscopy

**DOI:** 10.1021/acsnano.5c00591

**Published:** 2025-02-28

**Authors:** Andrea Auer, Franz J. Giessibl, Julia Kunze-Liebhäuser

**Affiliations:** † Institute of Physical Chemistry, University of Innsbruck, 6020 Innsbruck, Austria; ‡ Institute of Experimental and Applied Physics, University of Regensburg, 93053 Regensburg, Germany

**Keywords:** electrochemical scanning tunneling microscopy, atomic force microscopy, qPlus sensor

## Abstract

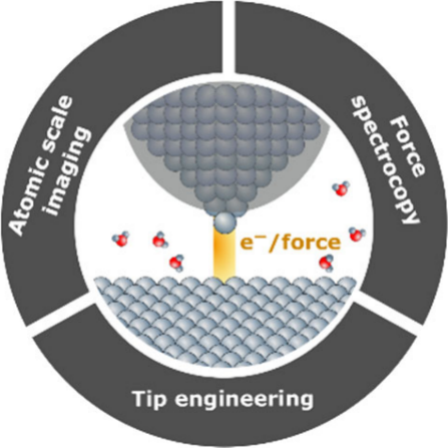

All electrochemical and electrocatalytic processes occur at the boundary between an electrode and an electrolyte. Progress in the field of electrochemistry requires a detailed microscopic understanding of these complex solid–liquid interfaces, making this a captivating field for in situ surface-sensitive microscopic techniques, such as scanning probe microscopy. In this Perspective, we outline the roadmap of electrochemical scanning probe microscopy and explore its most recent developments in fundamental research on interface characterization and electrocatalysis. Most importantly, we introduce the reader to the simultaneous operation of electrochemical scanning tunneling microscopy and force microscopy using a qPlus sensor, highlighting its potential to provide high precision, enhanced flexibility and versatility, particularly as a combined approach to interface characterization. Additionally, we identify key future opportunities and challenges.

High-resolution scanning probe microscopy has enabled the direct visualization of atoms and is thus—since its early stages—an important technique throughout multidisciplinary fields, ranging from physics, chemistry and engineering to biology and life sciences. Already shortly after the development of scanning tunneling microscopy (STM)^1^ and later atomic force microscopy (AFM),^2^ where mostly the solid-vacuum interface was studied, the technique has quickly been adapted for electrochemistry. First investigations in the field included imaging of Pt and Au electrode surfaces in air, which have been emersed from the electrolyte after electrochemical treatment.^3^ STM rapidly moved toward true in situ imaging, where it was performed in nonaqueous liquids, such as oil and liquid nitrogen,^4^ before Sonnenfeld and Hansma showed for the first time that it also works well in aqueous electrolyte solutions.^5^ Since only two electrodes—the sample and the tip—were used, full potential control was not guaranteed in these experiments. Therefore, Siegenthaler et al. introduced a reference electrode in the electrochemical (EC-) STM cell, which allowed to control the potentials of the sample and the tip independently with a bipotentiostat.^6,7^ This circuit was then extended by Itaya et al.^7^ and Wiechers et al.,^8^ who added a counter electrode as a fourth electrode to the setup in order to measure the Faradaic currents in the system. This last step toward the four-electrode configuration, as it is used nowadays has established EC-STM as a true in situ microscopic technique for electrochemical experiments. It is imperative to note that the STM tip is conductive. Therefore, the tip must be insulated by a coating; otherwise, a high Faraday current will flow. In contrast, for AFM generally less adaptations and precautions need to be taken into consideration for true in situ imaging and the integration of an external potentiostat with an ambient AFM is sufficient to yield an in situ EC-AFM configuration. Therefore, shortly after the first atomically resolved AFM images in air and liquid,^9^ in situ EC-AFM was demonstrated by Gewirth et al.^10^

The principle of both SPM techniques relies on probing a defined interaction between a sharp tip and a sample surface (Figure 1). In STM, the interaction is the tunneling current, which is highly sensitive to the tip–sample distance. This enables atomic-scale spatial resolution through imaging of the conductance, which corresponds to the local density of states (LDOS), at the Fermi level. AFM, in contrast, detects local forces, specifically the spatially confined interaction between the atoms at the tip apex and the sample surface.^11,12^ This interaction can be decomposed into multiple force components, including short-range, such as Pauli exclusion and chemical bonding forces, and long-range, such as van der Waals and electrostatic forces.^12^ In liquid environments, additional forces such as hydration or solvation forces and viscous damping can further complicate the tip–sample interaction.^13^ The overlap of these forces can pose challenges for achieving high-resolution imaging. However, early studies also demonstrated that performing AFM in liquid can reduce (long-range) background forces, which results in more stable imaging conditions overall.^14^

**Figure 1:**
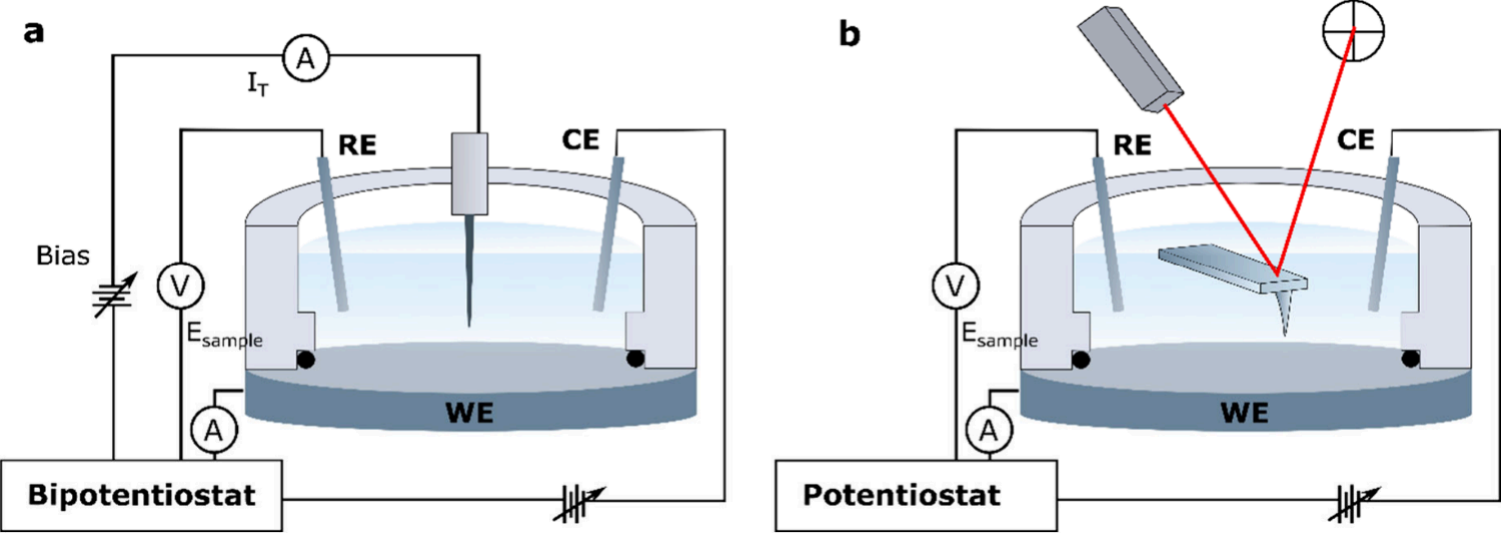
(a) Scheme of the electrochemical STM principle. This technique measures the tunneling current (I_T_) between a sharp, insulated tip and a conductive sample (working electrode, WE) immersed in an electrolyte. (b) Scheme of the electrochemical AFM principle. Here, the sharp tip interacts with the sample through a combination of short- and long-range forces. These interactions are quantified by measuring the deflection of the cantilever, enabling both surface imaging and force measurements. Both setups include an in situ electrochemical cell and a (bi)potentiostat.

In the case of electrochemical application, the microscopes are equipped with an in situ electrochemical cell and a (bi)potentiostat, as schematically drawn in Figure 1. Electrochemical SPM, including both EC-STM and EC-AFM, has fundamentally advanced our understanding of electrode structure and morphology by providing atomically resolved images of various electrochemical processes. These include, but are not limited to, surface reconstructions, chemisorption, metal deposition, phase transitions, and, more recently, electrocatalytic surface reactions.

This Perspective is divided into three sections. The first explores recent innovations in EC-SPM, addressing both fundamental interfacial studies and more applied electrocatalysis research. It also contextualizes the development of a combined EC-AFM/STM in the light of other hybrid and multimodal SPM approaches. The second introduces the principle of simultaneously operating STM and AFM using a qPlus sensor, highlighting its potential to provide unprecedented insights into the nanoscale structure of the electrified solid–liquid interface. The third section discusses key opportunities, challenges, and future prospects, focusing on the qPlus-based EC-SPM as a combinatorial characterization approach. Together, these sections aim to underscore the role of emerging SPM methodologies in tackling the complexities of electrochemical interfaces and driving innovation in the field.

## Innovation and Progress in the Field of Electrochemical Scanning Probe Microscopy

### Scanning Tunneling Microscopy

Metal surfaces are well-known to reconstruct in order to minimize their surface energy, where the extent of reconstruction is often strongly dependent on the work function. The electrochemical environment offers the unique possibility to systematically vary the electronic state of a surface through application of a potential and the effect of adsorbed electrolyte species.^15−17^ The ability of EC-STM to provide real-space structural information on the state of the surface during a systematic variation of the electrochemical conditions has thus yielded important information on the restructuring of metal surfaces.

Adsorption of electrolyte species is central to electrochemical reactions, particularly the specific adsorption of anions, which leads to potential-dependent ordered adlayers.^15,16,18,19^ These surface dynamics—diffusion, adsorbate interactions, and structural transitions—are critical to electrocatalysis. Early EC-STM studies correlated diffusion kinetics with potential and anion type, but higher temporal resolution was needed. The development of high-speed STM^20,21^ in the late 2000s enabled visualization of rapid surface processes, such as the millisecond-scale dynamic phase behavior of adsorbed CO on Pt(111).^22^ However, studies of low-coverage, disordered adlayers and weakly adsorbed species remain limited.^18^

**Figure 2:**
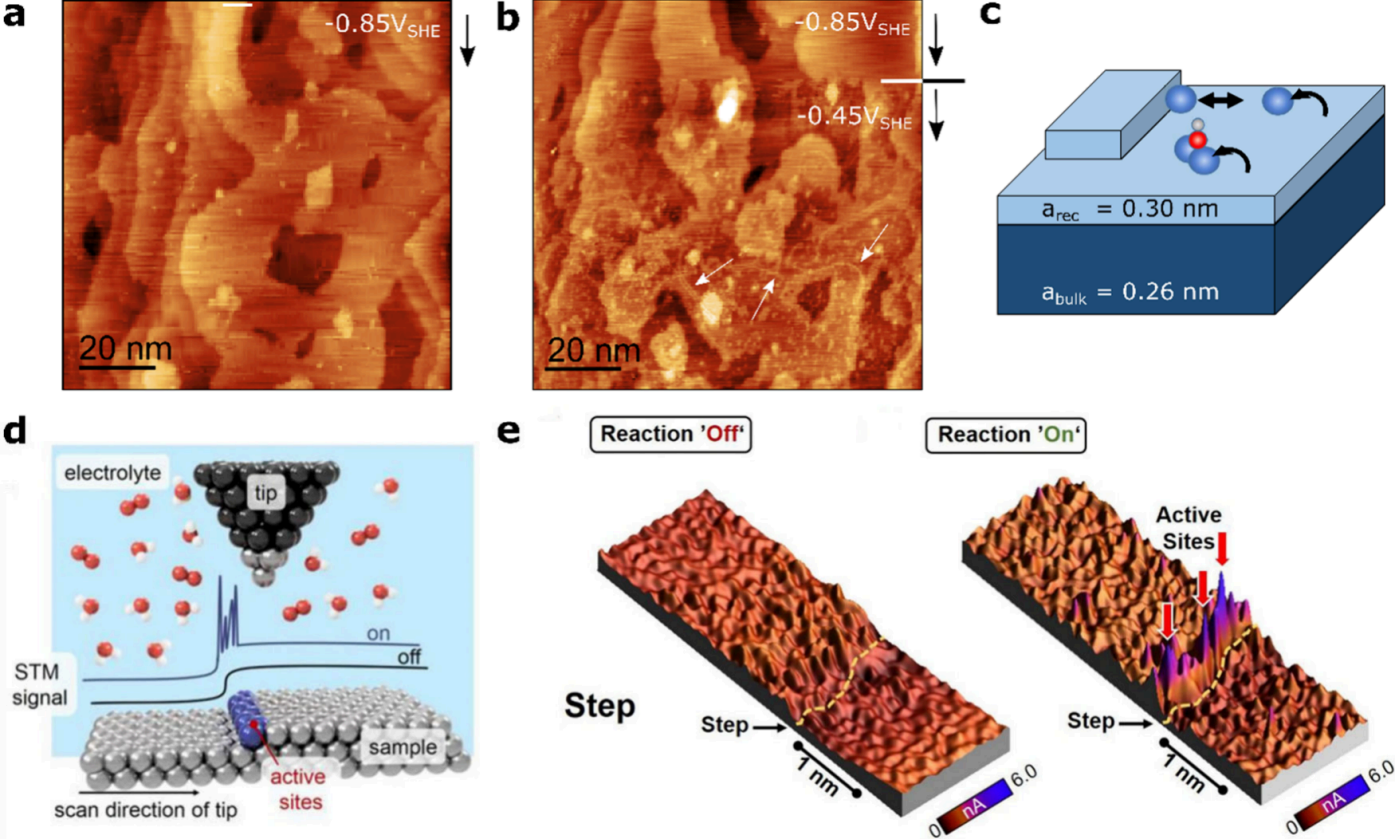
(a-c) Direct EC-STM visualization of Cu(111) under CO oxidation reaction conditions: In-situ images of (a) metallic Cu and (b) during a potential step into the CO oxidation regime where surface reconstruction takes place. (c) Schematic representation of the restructuring. Reprinted with permission from ref (23). Copyright 2020 The Author(s), under exclusive license to Springer Nature Limited. (d-e) Revealing catalytically active sites by noise-EC-STM: (d) Schematic representation of the concept where the tunneling-current noise is higher above the active sites. (e) Noise-EC-STM of a graphite electrode under hydrogen evolution reaction (HER) for reaction “off” and “on” condition. An increased noise level is observed at the step for the reaction “on”. Reprinted with permission under a Creative Commons CC BY License from ref (28). Copyright 2023 The Author(s).

With the growth of electrocatalysis, EC-STM has become an essential tool for characterizing catalysts at the atomic scale under reaction conditions. EC-STM was therefore employed to reveal the structural evolution of electrode surfaces under operating conditions. For example, visualization of the Cu(111) catalyst surface during the CO oxidation reaction revealed the quasi-reversible formation of cluster-like protrusions with under-coordinated Cu atoms to be essential for the observed activity (Figure 2a-c).^23^ This emphasizes the necessity to accurately determine the structure of the active catalyst surface. Additionally, noise analysis in the tunneling current has been proposed by Bandarenka et al.^24^ and further utilized by Granozzi et al.^25−27^ as a method to identify active sites at the solid-electrolyte interface, offering a new approach to locally evaluate catalytic activity (Figure 2d-e).

### Atomic Force Microscopy

EC-AFM has enabled the acquisition of high-resolution images of various surface processes including adsorption, oxidation, corrosion and electrocatalysis.^29^ However, one of the major advantages of AFM is that, unlike STM, it can be operated with little to no interference from Faradaic currents coming from the sample and/or the tip. Consequently, AFM can facilitate the study of systems characterized by high electrochemical activity.^29^ Both static and dynamic modes of AFM have been applied to electrochemical interface studies. Especially frequency modulation AFM (FM-AFM), where the cantilever oscillates at its resonance frequency and a fixed amplitude is maintained, is very promising for high spatial resolution and force sensitivity. The attainment of true atomic resolution in water by means of FM-AFM by the pioneering work of Fukuma et al.^30^ in 2005 represents a significant advancement in the field, enabling the nondestructive investigation of atomic-scale phenomena that occur at the solid–liquid interface. Only a few years later, Fukui et al.^31,32^ extended FM-AFM to electrochemical systems, leading to the development of electrochemical frequency modulation AFM (EC-FM-AFM). Yet, studies in electrolyte solution and with potential control remain extremely limited.

**Figure 3:**
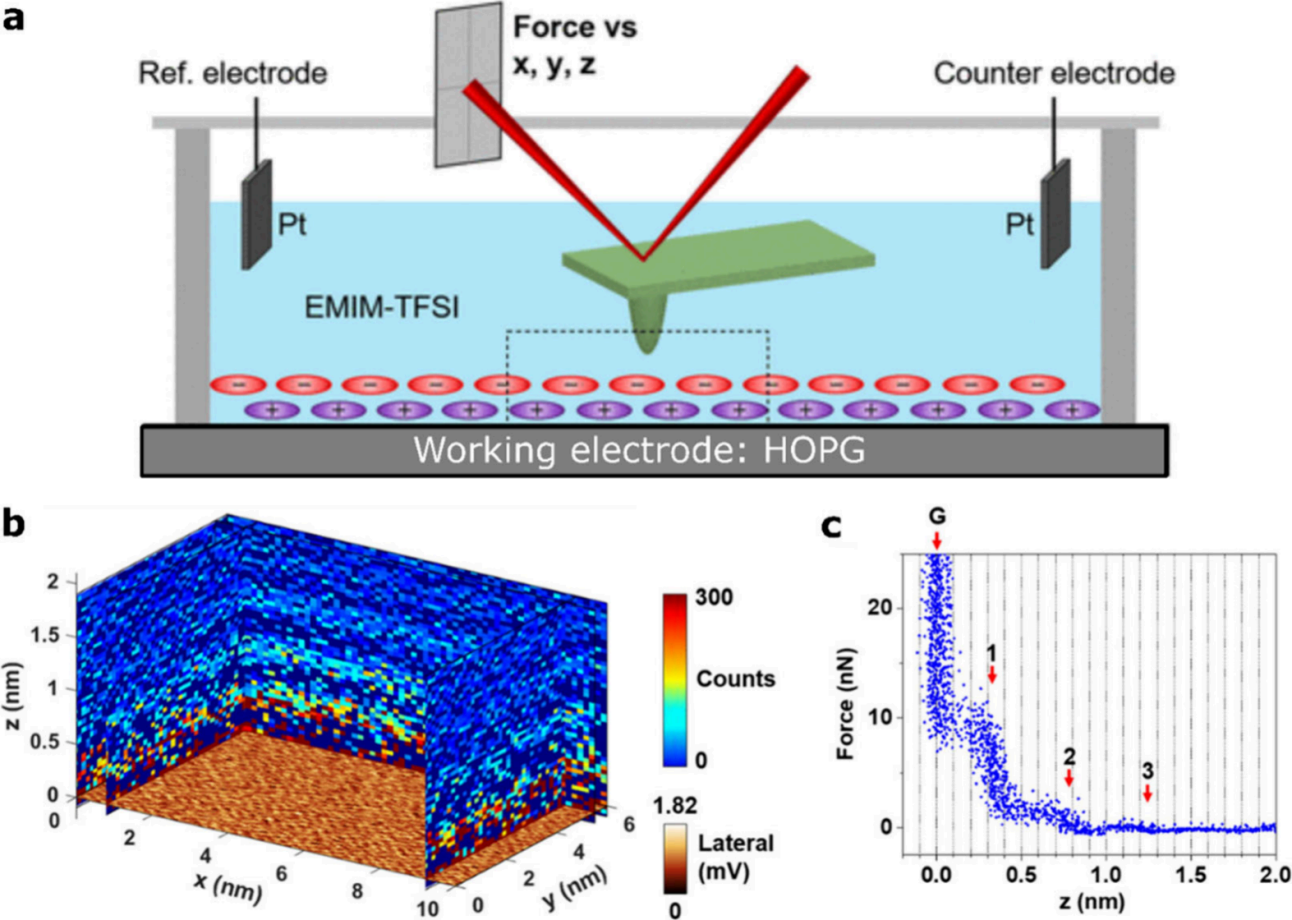
(a) EC-3D-AFM imaging at the HOPG-ionic liquid interface. (b) 3D image of one area of the electrode–electrolyte interface, consisting of the x–y lateral image of the HOPG surface and the x–z and y–z count maps revealing the EDL of EMIM-TFSI. (c) Overlap of 50 force–distance curves at single x-position, where the red arrows mark the positions of the graphite surface (G) and the first three electrolyte layers. Reprinted from ref (33). Copyright 2020 American Chemical Society.

While EC-STM predominantly focuses on the structure and morphology of surfaces, dynamic AFM has resolved nanoscale electrolyte structuring through force–distance measurements. ^e.g.,^(34−39) More recently, liquid-phase 3D-AFM has been developed and was used to image electrified solid–liquid interfaces and their lateral heterogeneity. ^e.g.,^(40−47) Using either amplitude or frequency modulation, the tip–sample interaction force is simultaneously imaged in the x, y- and *z*-directions to create 3D maps of the solid–liquid interface structure. Due to its high sensitivity, this technique has the capacity to resolve both the atomic structure of the solid surface and the molecular arrangement of the liquid electrolyte.^42^ However, electrochemical 3D-AFM studies remain limited, with - to the best of our knowledge - only one reported investigation on the solid–ionic liquid interface (Figure 3),^33^ revealing potential-dependent layering at the electrical double layer, that change with the electrode potential.

### Hybrid and Multimodal Approaches in EC-SPM

Various hybrid approaches in EC-SPM have demonstrated the potential for complementary microscopic characterization of the solid–liquid interface. For example, scanning electrochemical microscopy (SECM) integrates nanoelectrodes with AFM probes to facilitate localized electrochemical detection while deconvoluting topographic contributions. Additionally, liquid-phase (EC)-AFM with electrical signal detection has enabled localized (surface) potential measurements in both contact^48^ and noncontact modes.^49^

Recent innovations in EC-SPM, including high-speed STM, noise-EC-STM, and liquid-phase 3D-AFM, have significantly expanded our ability to characterize electrochemical interfaces. However, at the atomic scale, these techniques remain largely confined to either conductance- or force-based properties. A combined EC-STM/AFM system provides a synergistic platform to correlate topography with force interactions and local electronic structure properties, offering new insights into adsorption phenomena, reaction intermediates, and surface reconstructions under in situ conditions. This setup extends upon previous hybrid techniques by allowing AFM to act as feedback, while the conductive tip provides electrical signal detection, thereby also enabling established functionalities such as SECM.

The ability to simultaneously operate STM and AFM represents a crucial step toward a more complete understanding of electrochemical interfaces. This is particularly relevant in electrocatalysis, where the interplay between surface electronic states, adsorbate interactions, and local force distributions governs reactivity.

## Simultaneous Operation of EC-STM and AFM with a qPlus Sensor

The field of electrochemical surface science has historically drawn inspiration from cutting-edge UHV-based analytical techniques and integrated them into electrochemistry.^50^ This approach is also exemplified by the combination of electrochemical STM and its simultaneous operation with force microscopy using a qPlus sensor. The qPlus sensor, initially developed on the basis of a quartz tuning fork, is now custom built.^12^ It functions as a self-sensing device, utilizing the piezoelectric effect of quartz, and enables the operation of both STM and AFM in parallel. The development of the qPlus sensor has led to the achievement of subatomic spatial resolution in its AFM channel, surpassing the resolution of STM under UHV conditions. AFM with a qPlus sensor is typically operated in frequency-modulation detection mode, wherein the oscillating cantilever’s frequency is altered by the gradient of the force that acts between the tip and the sample.^12^ FM-AFM in a low-temperature microscope has emerged as the method that provides (sub)atomic spatial resolution and force spectroscopy with subpiconewton sensitivity. It operates in true noncontact mode, thereby enabling simultaneous AFM and STM measurements.^12,51^

The application of the qPlus sensor has strongly advanced high-precision measurements of surface processes under UHV conditions. More recently, it has been extended to ambient conditions^52−55^ and liquids.^56,57^ In EC-STM, an insulated tip with a small conductive part at the tip apex is immersed in an electrolyte, as shown in Figure 1.^19^ Traditional AFM uses silicon cantilevers that are too small to allow mounting a typical EC-STM tip, thus requires more complex modifications. However, the qPlus sensor is large and allows to mount large and heavy tips. Therefore, an ambient qPlus AFM^56^ was converted into a combined electrochemical STM/AFM.^58^

Expanding the application of the qPlus sensor from UHV to electrochemical environments requires careful consideration of the differences in tip–sample interactions. In FM-AFM, the cantilever is oscillated at its resonance frequency (*f*_0_). As the tip approaches a surface, the force between the tip and the sample (*F*_ts_) induces a shift of the cantilever resonance frequency (Δ*f*). If the oscillation amplitude is small enough to assume that over a whole oscillation cycle the force gradient stays constant, the frequency shift is proportional to the weighted average force gradient and given by^59^
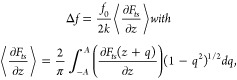
where *k* denotes the spring constant and *A* the amplitude of the cantilever.^60^ This relationship highlights the direct sensitivity of FM-AFM to the local force gradient, which is particularly relevant for short-range interactions.

Unlike FM-AFM, which measures resonance frequency shifts, amplitude modulation AFM (AM-AFM) operates by driving the cantilever at a fixed frequency near resonance and detecting changes in oscillation amplitude and phase due to tip–sample interactions.^61^ If the amplitude is large, intermittent contact with the surface during each oscillation cycle can occur. This is deliberately used in tapping mode AFM, a type of AM-AFM, in which the tip contacts the surface and detaches from the sample during each oscillation cycle.^62^ In UHV, AM-AFM is generally less sensitive than FM-AFM, because the high Q-factor of the cantilever leads to a long amplitude response time, which limits its ability to track rapid force variations at the atomic scale. However, in liquids, where strong damping drastically lowers Q, AM-AFM can provide stable imaging conditions and atomic scale contrast. Thus, both FM-AFM and AM-AFM have demonstrated the ability to achieve atomic and molecular resolution in ambient and liquid environments,^61,63^ and, to the best of our knowledge, there is still no quantitative evidence proving the intrinsic superiority in terms of spatial resolution of one method over the other.

While AM-AFM is simpler to implement, more widely available commercially and more versatile for imaging both small and large (up to several μm^2^) scan areas,^61^ a key advantage of FM-AFM is its direct measurement of the frequency shift Δ*f*. This enhances sensitivity to short-range forces due to its proportionality to the average force gradient at small amplitudes and thereby minimizes tip-induced perturbations. FM-AFM also does not suffer from the mode hopping instability of AM-AFM^64,65^ and allows for the precise distinction between different interaction regimes. Additionally, FM-AFM provides a separate dissipation signal, as the excitation amplitude required to maintain a constant oscillation amplitude can be measured independently. This second feedback loop enables the differentiation between energy-conserving (conservative) and energy-dissipative interactions, whereas AM-AFM does not fully disentangle conservative and dissipative forces. This can be particularly useful in electrochemical environments, where insights into the local site-specific viscosity variations at the interface may be related to electrochemical reactivity.^66,67^

**Figure 4:**
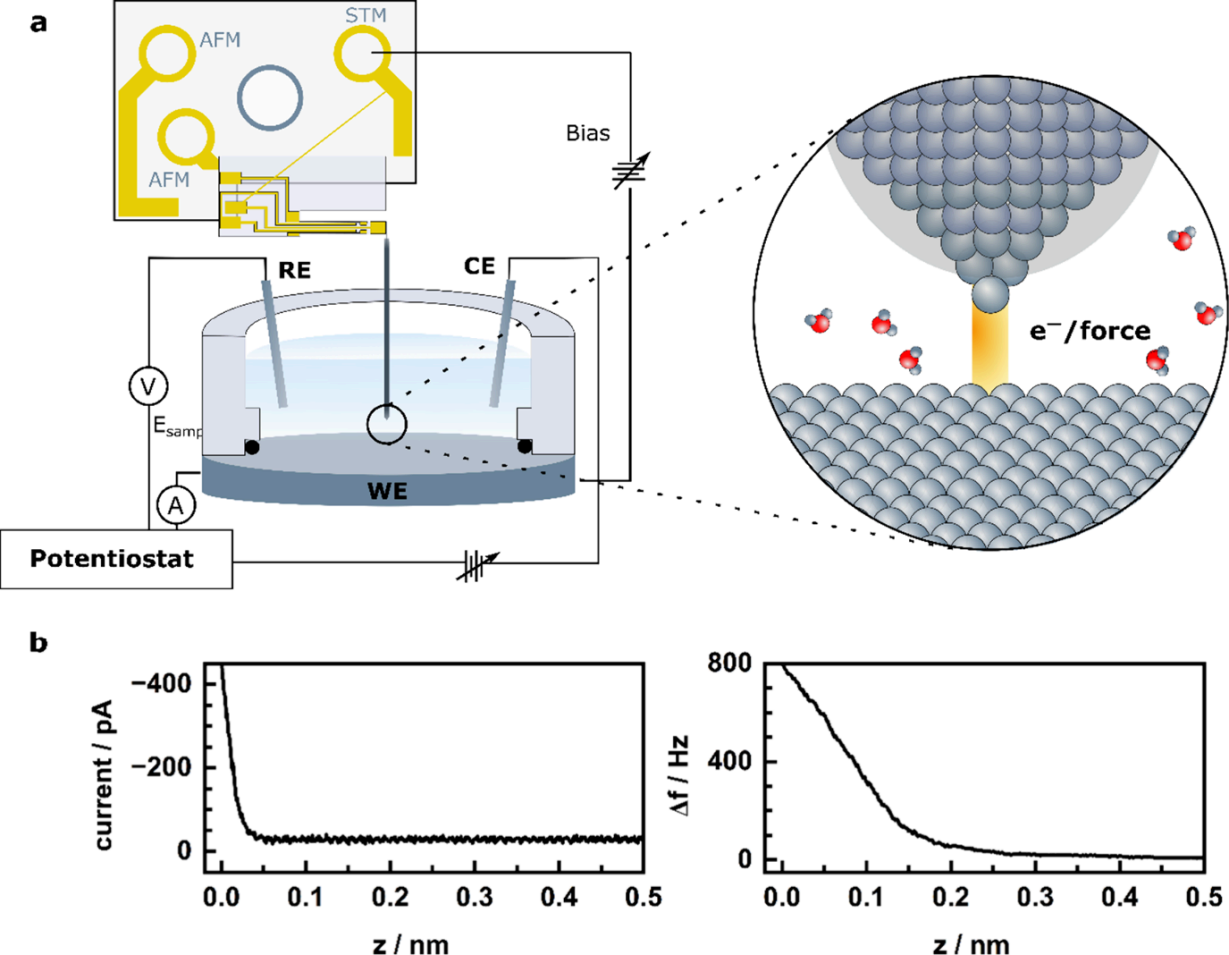
Combined EC-STM/AFM with a qPlus sensor. (a) Scheme of the working principle and (b) simultaneous z-spectroscopy measurement showing the tunneling current (STM channel) and the frequency shift (AFM channel) versus the relative distance z.

The electrochemical STM/AFM system based on a qPlus sensor is schematically shown in Figure 4a. A parallel measurement of the EC-STM and AFM channels as a function of the tip height (z-spectroscopy measurement) is shown in Figure 4b. While the tunneling current shows the expected exponential decay down to the leakage current, the EC-AFM signal shows a very different distance dependence, demonstrating the possibility to independently and simultaneously measure the two signals. Positive frequency shifts are typically used in liquids, suggesting that the tip experiences a net repulsive force, which differs from the attractive-regime operation often emphasized as true noncontact mode in UHV studies. Importantly, however, the presence of (strong) hydration layers means that even in a highly repulsive regime, the tip may still be separated from the surface, preventing direct mechanical contact. This is also illustrated in Figure 4b, where already at a z position of 0.2 nm a significant positive Δ*f* signal appears, whereas tunneling current is not measurable until below a relative distance of 0.05 nm.

**Figure 5:**
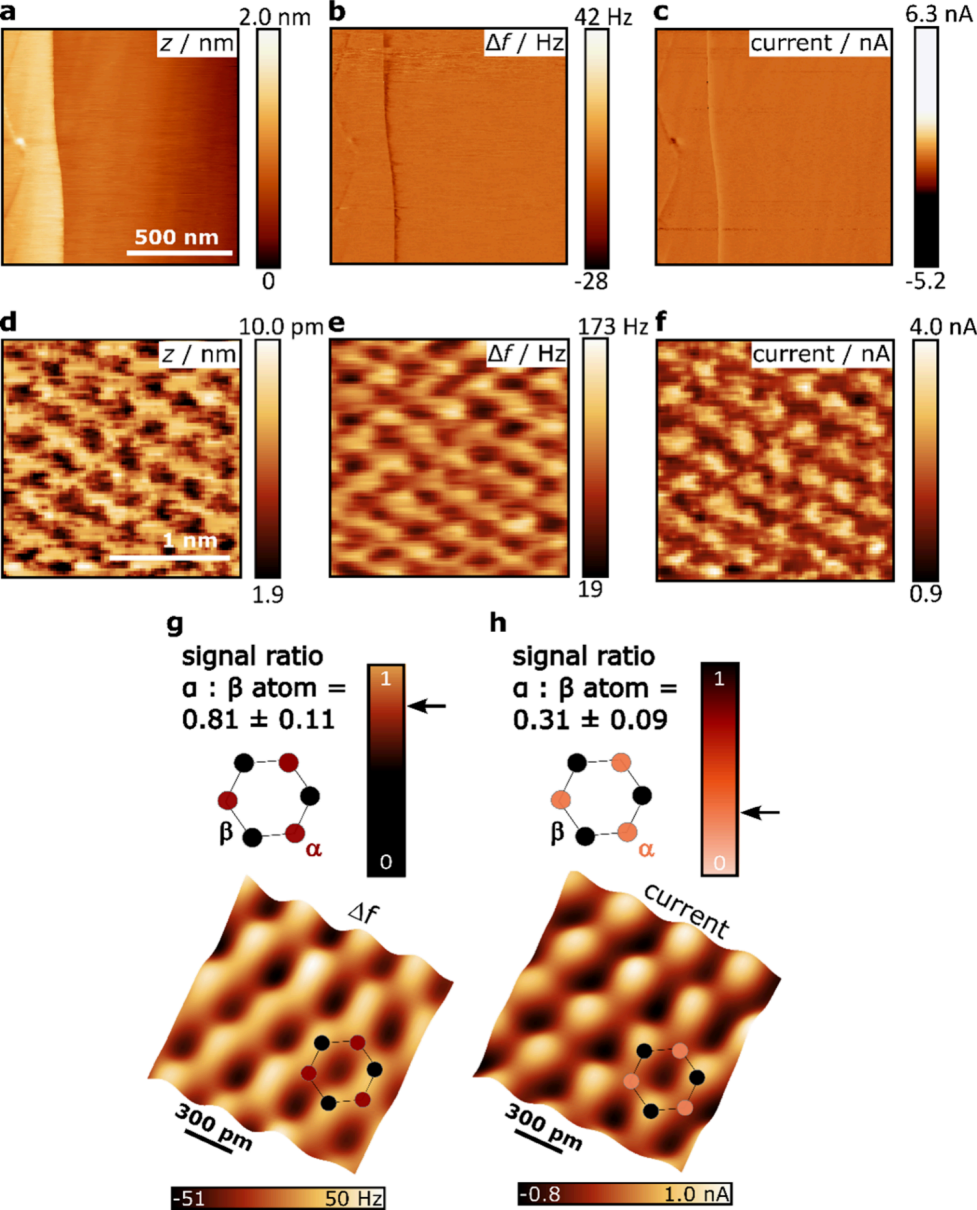
Simultaneous electrochemical AFM/STM images of freshly cleaved HOPG. Large scan images of the electrode surface in the (a) topographic (*z*), (b) frequency shift (Δ*f*) and (c) tunneling current channel. (d-f) Atomically resolved images taken in quasi-constant height mode with an amplitude of 50 pm. Evaluated signal contrast of the α and β atom with respect to the hollow site and reconstructed images by inverse 2D fast Fourier transformation of the frequency shift and tunneling current images. Reprinted from ref (58), with the permission of AIP Publishing. Copyright 2023 The Author(s).

We were recently able to show that high-resolution, simultaneous operation of STM and AFM is possible in ambient, in general, and under electrochemical conditions, in particular.^58^ This was achieved by atomically resolving a graphite electrode in sulfuric acid under potential control (see Figure 5).^58^ The large area images (Figure a-c) taken in AFM feedback with an applied bias between tip and sample, show large terraces separated by a single step, which is visible in all channels. Atomic resolution of the graphite surface is shown in Figure 5d-f. Most strikingly, a distinct difference can be observed in the frequency shift (Δ*f*) and the tunneling current images: While in the STM image, only every second atom, the β atom, of the hexagonal lattice is visible due to the different electronic structure of the neighboring carbon atoms, we show that AFM can resolve both the α and β atoms of the graphite structure as a result of the total charge interaction with the tip under electrochemical conditions. To quantify the observed difference in contrast, the signal ratio between the α and β atom with relative to the hollow site is shown in Figure 5g-h. The signal intensity over the α and β sites is determined, which is the difference between the signal at the α (or β) site and the minimal value of the signal, which occurs at the center of the hexagons. Then the signal ratio α:β is calculated, which is the ratio between the signal intensity at the α site and the signal intensity at the β site. While the α atom shows a similar intensity as the β atom in the Δ*f* image (with α:β ≈ 0.8), it appears at a lower intensity in the current image (with α:β ≈ 0.3).^58^ Thus, similarly to what was shown under UHV conditions,^68^ EC-STM measures the conductance at the Fermi level, whereas EC-FM-AFM (in the repulsive regime) reflects the total charge density of the sample. This opens up a wealth of opportunities to gain new insights into surface processes associated with a change in charge density, such as adsorption, intercalation and oxidation.

## Opportunities, Challenges and Summary

Over the past decades, electrochemical SPM has led to significant advances in the field’s comprehension of interfacial properties and structure at the atomic, molecular and nanoscale level.^19,29,69^ The development of simultaneous operation of AFM and STM offers new possibilities. The following section outlines the main future opportunities as well as the challenges that need to be addressed.

### 1 Atomic Scale Imaging

As previously outlined, STM is sensitive to the LDOS of the sample at the Fermi level, whereas AFM measures the interaction between the tip and the sample. As a result, AFM is more sensitive to the total charge density of a sample. Under UHV conditions, therefore simultaneous STM/AFM has been found ideal for elucidating the structural properties of non- or semiconducting oxides and their defect-related surface chemistry.^12^ In general, STM alone is often unable to unambiguously identify defects, while AFM can provide a more precise determination of their physical and structural properties.^70^ This is similarly true for adsorbed surface species, where the distribution of charge density near the Fermi level dominates the observed STM contrast. This is highly important for electrochemical systems as demonstrated by the case of one of the most classical ordered structure in electrochemistry, namely the  structure on a Au(111) surface in sulfuric acid. While multiple adsorption configurations have been proposed in the past four decades mostly based on STM imaging, the structure remained ambiguous. Only in 2020 with the help of theoretical calculations, electrochemical infrared and Raman spectroscopy, high-resolution STM could elucidate the adlayer structure.^71^ EC-AFM complemented by STM offers a powerful alternative for achieving a more unequivocal structural elucidation of complex adsorbate as well as defect structures at electrode surfaces. Furthermore, the electrical detection of the qPlus sensors renders them highly appealing for electrochemical investigations, as the necessity for a laser is eliminated, enabling the study of more complex processes, such as photo(electro)catalytic reactions, without the risk of interference.

A commonly cited advantage of AFM is its capacity to image nonconductive surfaces, such as insulators. While this may appear irrelevant for electrochemical applications, where conductivity of the sample is essential for its reactivity, it has been demonstrated that the image quality of oxides or (electrochemically) oxidized surfaces is often constrained in EC-STM and the contrast is highly ambiguous.^72^ Simultaneous EC-AFM/STM can overcome these restrictions, providing new atomic scale insights on semiconducting or thin oxide electrode surfaces by measuring both the conductance at the Fermi level and the total charge interaction.

### 2 Force Spectroscopy

The stiffness of the qPlus sensors allows the use of very small amplitudes in frequency modulation detection mode, thereby ensuring high sensitivity to short-range forces. This property facilitates the acquisition of force spectra with minimal or no downward force, thus preventing damage or deformation of the soft double layer.^58^ The qPlus-based EC-AFM offers the capability to observe the intricate interfacial organization of solvent molecules, such as water at the electrochemical HOPG-liquid interface.^58^ In order to shed light on heterogeneity in the lateral (solvent) structure, it is necessary to incorporate a true 3D-AFM imaging mode.^33^ In addition, the precise role of the tip functionality remains to be fully elucidated, which requires precise and above all knowledge-based tip engineering.

### 3 Tip Engineering

One key part of a force sensor is the probe tip. Commercially available, micromachined Si cantilevers typically have sharp etched Si tips that are covered by a native oxide. In contrast, qPlus sensors often have metal tips, that are intentionally terminated with a single CO molecule^73^ or copper oxide (CuO_*x*_)^74^ under low-temperature and UHV conditions. In both CO terminated tips and CuO_*x*_ tips, the oxygen atom acts as an inert probe termination. The question arises if there exists an equally controlled method for fabricating tips with a precise geometry and chemical (inert) nature that work in liquid and at room temperature. As Voïtchovsky notes in his viewpoint,^75^ the structural and chemical details of the nanoscale tip utilized for ambient measurements are typically not known and complete characterization of the tip is difficult, time-consuming, and can even be destructive. Furthermore, subsequent contamination can occur after characterization, typically due to organics in the ambient. The problem is amplified in liquids, where the hydration of the tip plays a critical role in both high-resolution imaging^76^ and force spectroscopy measurements.^77,78^ Consequently, a multitude of well-designed studies are necessary to not only reliably control the shape of the tip apex and its chemistry but also to fundamentally understand the precise role of the tip and ultimately allow for a more quantitative molecular-level characterization of the entire solid–liquid interface. In this context, chemical modification of SPM tips presents a promising approach. While the STM as well as AFM tip can be easily functionalized in UHV by transferring a single molecule from the surface, resulting in unprecedent spatial resolution,^73,79^ studies in ambient conditions for STM^80^ and even in liquid for AFM^44^ have shown that forming a stable self-assembled (mono)layer on the tip can enhance chemical contrast by selectively recognizing specific species and functional groups. Further exploration of this longstanding yet underutilized concept could offer a rigorous way to control the tip chemistry and, to some extent, even its shape, particularly in the context of high-resolution imaging.

In this regard, the qPlus sensor represents a promising approach due to its flexibility and versatility in tip selection. In the combined EC-STM/AFM setup, the tip and the sample can be electrochemically controlled independently of each other. Thus, the tip chemistry can also be modified in situ by simply altering the potential. However, this approach directly implies a change in bias, which can result in a possibly interfering electric field between the tip and the sample. It is imperative to be aware of the effects of this field, which have the capacity to alter the intrinsic structural properties at the electrified solid–liquid interface.

In summary, electrochemical SPM has largely contributed to a more comprehensive understanding of heterogeneity in both surface structure and morphology, as well as interfacial electrochemical response. It is this local structural (and chemical) variability of electrode surfaces that often determines the overall activity of functional energy materials. Therefore, further development of instrumentation and the exploration and combination of SPM-based methods as demonstrated by the combination of EC-STM with force spectroscopy are key to disentangling structure–activity relationships at the relevant nanoscale. In this regard, local multimodal characterization is emerging as an increasingly important path toward an in-depth understanding of both fundamental interface research and practical electrochemistry. Pragmatically, the current state-of-the-art can be summarized as follows: in liquid-phase (EC)-SPM it is possible to resolve some types of surfaces with atomic resolution using some kind of tips and instrumentation. Yet, when attempting to generalize these capacities, significant challenges emerge. The qPlus-based electrochemical STM/AFM offers an unconventional platform to address and overcome these challenges, where further instrument development, tip engineering, refinement of existing SPM-based approaches, and its integration with other techniques might lead to better atomic control and a better understanding of solid–liquid interfaces at the nanoscale.

## References

[ref1] Binnig G.; Rohrer H.; Gerber Ch.; Weibel E. Surface Studies by Scanning Tunneling Microscopy. Phys. Rev. Lett. 1982, 49 (1), 57–61. 10.1103/PhysRevLett.49.57.

[ref2] Binnig G.; Quate C. F.; Gerber Ch. Atomic Force Microscope. Phys. Rev. Lett. 1986, 56 (9), 930–933. 10.1103/PhysRevLett.56.930. 10033323

[ref3] Gómez J.; Vázquez L.; Baró A. M.; Garcia N.; Perdriel C. L.; Triaca W. E.; Arvia A. J. Surface Topography of (100)-Type Electro-Faceted Platinum from Scanning Tunnelling Microscopy and Electrochemistry. Nature 1986, 323 (6089), 612–614. 10.1038/323612a0.

[ref4] Drake B.; Sonnenfeld R.; Schneir J.; Hansma P. K.; Slough G.; Coleman R. V. Tunneling Microscope for Operation in Air or Fluids. Rev. Sci. Instrum. 1986, 57 (3), 441–445. 10.1063/1.1139208.

[ref5] Sonnenfeld R.; Hansma P. K. Atomic-Resolution Microscopy in Water. Science (1979) 1986, 232 (4747), 211–213. 10.1126/science.232.4747.211. 17780805

[ref6] Lustenberger P.; Rohrer H.; Christoph R.; Siegenthaler H. Scanning Tunneling Microscopy at Potential Controlled Electrode Surfaces in Electrolytic Environment. J. Electroanal Chem. Interfacial Electrochem 1988, 243 (1), 225–235. 10.1016/0022-0728(88)85043-5.

[ref7] Itaya K.; Tomita E. Scanning Tunneling Microscope for Electrochemistry - a New Concept for the in Situ Scanning Tunneling Microscope in Electrolyte Solutions. Surf. Sci. 1988, 201 (3), L507–L512. 10.1016/0039-6028(88)90489-X.

[ref8] Wiechers J.; Twomey T.; Kolb D. M.; Behm R. J. An In-Situ Scanning Tunneling Microscopy Study of Au (111) with Atomic Scale Resolution. J. Electroanal Chem. Interfacial Electrochem 1988, 248 (2), 451–460. 10.1016/0022-0728(88)85106-4.

[ref9] Manne S.; Butt H. J.; Gould S. A. C.; Hansma P. K. Imaging Metal Atoms in Air and Water Using the Atomic Force Microscope. Appl. Phys. Lett. 1990, 56 (18), 1758–1759. 10.1063/1.103091.

[ref10] Manne S.; Hansma P. K.; Massie J.; Elings V. B.; Gewirth A. A. Atomic-Resolution Electrochemistry with the Atomic Force Microscope: Copper Deposition on Gold. Science (1979) 1991, 251 (4990), 183–186. 10.1126/science.251.4990.183. 17836948

[ref11] Bian K.; Gerber C.; Heinrich A. J.; Müller D. J.; Scheuring S.; Jiang Y. Scanning Probe Microscopy. Nature Reviews Methods Primers 2021, 1 (1), 36 10.1038/s43586-021-00033-2.

[ref12] Giessibl F. J. The qPlus Sensor, a Powerful Core for the Atomic Force Microscope. Rev. Sci. Instrum. 2019, 90 ( (1), ). 10.1063/1.5052264. 30709191

[ref13] Israelachvili J. N. Intermolecular and Surface Forces; Elsevier, 2011. 10.1016/C2009-0-21560-1.

[ref14] Ohnesorge F.; Binnig G. True Atomic Resolution by Atomic Force Microscopy Through Repulsive and Attractive Forces. Science (1979) 1993, 260 (5113), 1451–1456. 10.1126/science.260.5113.1451. 17739801

[ref15] Gewirth A. A.; Niece B. K. Electrochemical Applications of *in Situ* Scanning Probe Microscopy. Chem. Rev. 1997, 97 (4), 1129–1162. 10.1021/cr960067y. 11851445

[ref16] Magnussen O. M. Ordered Anion Adlayers on Metal Electrode Surfaces. Chem. Rev. 2002, 102 (3), 679–725. 10.1021/cr000069p. 11890754

[ref17] Kolb D. Reconstruction Phenomena at Metal-Electrolyte Interfaces. Prog. Surf. Sci. 1996, 51 (2), 109–173. 10.1016/0079-6816(96)00002-0.

[ref18] Magnussen O. M.; Groß A. Toward an Atomic-Scale Understanding of Electrochemical Interface Structure and Dynamics. J. Am. Chem. Soc. 2019, 141 (12), 4777–4790. 10.1021/jacs.8b13188. 30768905

[ref19] Nowicki M.; Wandelt K. Electrochemical Scanning Tunneling Microscopy. In Encyclopedia of Interfacial Chemistry; Elsevier, 2018; pp 108–128. 10.1016/B978-0-12-409547-2.13621-2.

[ref20] Tansel T.; Magnussen O. M. Video STM Studies of Adsorbate Diffusion at Electrochemical Interfaces. Phys. Rev. Lett. 2006, 96 ( (2), ). 10.1103/PhysRevLett.96.026101. 16486600

[ref21] Schitter G.; Rost M. J. Scanning Probe Microscopy at Video-Rate. Materials Today. 2008, 11, 40–48. 10.1016/S1369-7021(09)70006-9.

[ref22] Wei J.; Amirbeigiarab R.; Chen Y. X.; Sakong S.; Gross A.; Magnussen O. M. The Dynamic Nature of CO Adlayers on Pt(111) Electrodes. Angewandte Chemie - International Edition 2020, 59 (15), 6182–6186. 10.1002/anie.201913412. 31919982 PMC7187359

[ref23] Auer A.; Andersen M.; Wernig E. M.; Hörmann N. G.; Buller N.; Reuter K.; Kunze-Liebhäuser J. Self-Activation of Copper Electrodes during CO Electro-Oxidation in Alkaline Electrolyte. Nat. Catal 2020, 3 (10), 797–803. 10.1038/s41929-020-00505-w.

[ref24] Pfisterer J. H. K.; Liang Y.; Schneider O.; Bandarenka A. S. Direct Instrumental Identification of Catalytically Active Surface Sites. Nature 2017, 549 (7670), 74–77. 10.1038/nature23661. 28880284

[ref25] Kosmala T.; Baby A.; Lunardon M.; Perilli D.; Liu H.; Durante C.; Di Valentin C.; Agnoli S.; Granozzi G. Operando Visualization of the Hydrogen Evolution Reaction with Atomic-Scale Precision at Different Metal–Graphene Interfaces. Nat. Catal 2021, 4 (10), 850–859. 10.1038/s41929-021-00682-2.

[ref26] Lunardon M.; Kosmala T.; Durante C.; Agnoli S.; Granozzi G. Atom-by-Atom Identification of Catalytic Active Sites in Operando Conditions by Quantitative Noise Detection. Joule 2022, 6 (3), 617–635. 10.1016/j.joule.2022.02.010.

[ref27] Lunardon M.; Kosmala T.; Ghorbani-Asl M.; Krasheninnikov A. V.; Kolekar S.; Durante C.; Batzill M.; Agnoli S.; Granozzi G. Catalytic Activity of Defect-Engineered Transition Me Tal Dichalcogenides Mapped with Atomic-Scale Precision by Electrochemical Scanning Tunneling Microscopy. ACS Energy Lett. 2023, 8 (2), 972–980. 10.1021/acsenergylett.2c02599. 36816778 PMC9926491

[ref28] Schmidt T. O.; Haid R. W.; Gubanova E. L.; Kluge R. M.; Bandarenka A. S. Electrochemical Scanning Tunneling Microscopy as a Tool for the Detection of Active Electrocatalytic Sites. Top Catal 2023, 66 (15–16), 1270–1279. 10.1007/s11244-023-01807-6.

[ref29] Wang W.-W.; Yan H.; Gu Y.; Yan J.; Mao B.-W. In Situ Electrochemical Atomic Force Microscopy: From Interfaces to Interphases. Annual Review of Analytical Chemistry 2024, 51, 54 10.1146/annurev-anchem-061422. 38603469

[ref30] Fukuma T.; Kobayashi K.; Matsushige K.; Yamada H. True Atomic Resolution in Liquid by Frequency-Modulation Atomic Force Microscopy. Appl. Phys. Lett. 2005, 87 ( (3), ). 10.1063/1.1999856.

[ref31] Umeda K. I.; Fukui K. I. Observation of Redox-State-Dependent Reversible Local Structural Change of Ferrocenyl-Terminated Molecular Island by Electrochemical Frequency Modulation AFM. Langmuir 2010, 26 (11), 9104–9110. 10.1021/la904797h. 20146517

[ref32] Utsunomiya T.; Yokota Y.; Fukui K. Electrochemical Atomic Force Microscopy. In Compendium of Surface and Interface Analysis; Springer Singapore, 2018; pp 73–78. 10.1007/978-981-10-6156-1_13.

[ref33] Zhou S.; Panse K. S.; Motevaselian M. H.; Aluru N. R.; Zhang Y. Three-Dimensional Molecular Mapping of Ionic Liquids at Electrified Interfaces. ACS Nano 2020, 14 (12), 17515–17523. 10.1021/acsnano.0c07957. 33227191

[ref34] Zhang X.; Zhong Y. X.; Yan J. W.; Su Y. Z.; Zhang M.; Mao B. W. Probing Double Layer Structures of Au (111)-BMIPF6 Ionic Liquid Interfaces from Potential-Dependent AFM Force Curves. Chem. Commun. 2012, 48 (4), 582–584. 10.1039/C1CC15463J. 22109542

[ref35] Utsunomiya T.; Yokota Y.; Enoki T.; Fukui K. I. Potential-Dependent Hydration Structures at Aqueous Solution/Graphite Interfaces by Electrochemical Frequency Modulation Atomic Force Microscopy. Chem. Commun. 2014, 50 (98), 15537–15540. 10.1039/C4CC07093C. 25354540

[ref36] Rakov D. A.; Chen F.; Ferdousi S. A.; Li H.; Pathirana T.; Simonov A. N.; Howlett P. C.; Atkin R.; Forsyth M. Engineering High-Energy-Density Sodium Battery Anodes for Improved Cycling with Superconcentrated Ionic-Liquid Electrolytes. Nat. Mater. 2020, 19 (10), 1096–1101. 10.1038/s41563-020-0673-0. 32367080

[ref37] Gao Q.; Tsai W. Y.; Balke N. In Situ and Operando Force-Based Atomic Force Microscopy for Probing Local Functionality in Energy Storage Materials. Electrochemical Science Advances. John Wiley and Sons Inc February 1, 2022. 10.1002/elsa.202100038.

[ref38] Black J. M.; Zhu M.; Zhang P.; Unocic R. R.; Guo D.; Okatan M. B.; Dai S.; Cummings P. T.; Kalinin S. V.; Feng G.; Balke N. Fundamental Aspects of Electric Double Layer Force-Distance Measurements at Liquid-Solid Interfaces Using Atomic Force Microscopy. Sci. Rep 2016, 6. 10.1038/srep32389. PMC5009352 27587276

[ref39] Black J. M.; Walters D.; Labuda A.; Feng G.; Hillesheim P. C.; Dai S.; Cummings P. T.; Kalinin S. V.; Proksch R.; Balke N. Bias-Dependent Molecular-Level Structure of Electrical Double Layer in Ionic Liquid on Graphite. Nano Lett. 2013, 13 (12), 5954–5960. 10.1021/nl4031083. 24215396

[ref40] Umeda K.; Kobayashi K.; Minato T.; Yamada H. Atomic-Scale Three-Dimensional Local Solvation Structures of Ionic Liquids. J. Phys. Chem. Lett. 2020, 11 (4), 1343–1348. 10.1021/acs.jpclett.9b03874. 31990558

[ref41] Umeda K.; Zivanovic L.; Kobayashi K.; Ritala J.; Kominami H.; Spijker P.; Foster A. S.; Yamada H. Atomic-Resolution Three-Dimensional Hydration Structures on a Heterogeneously Charged Surface. Nat. Commun. 2017, 8 ( (1), ). 10.1038/s41467-017-01896-4. PMC5727385 29235462

[ref42] Fukuma T.; Garcia R. Atomic- and Molecular-Resolution Mapping of Solid-Liquid Interfaces by 3D Atomic Force Microscopy. ACS Nano. American Chemical Society December 26, 2018; pp 11785–11797. 10.1021/acsnano.8b07216. 30422619

[ref43] Fukuma T.; Ueda Y.; Yoshioka S.; Asakawa H. Atomic-Scale Distribution of Water Molecules at the Mica-Water Interface Visualized by Three-Dimensional Scanning Force Microscopy. Phys. Rev. Lett. 2010, 104 ( (1), ). 10.1103/PhysRevLett.104.016101. 20366372

[ref44] Benaglia S.; Uhlig M. R.; Hernández-Muñoz J.; Chacón E.; Tarazona P.; Garcia R. Tip Charge Dependence of Three-Dimensional AFM Mapping of Concentrated Ionic Solutions. Phys. Rev. Lett. 2021, 127 ( (19), ). 10.1103/PhysRevLett.127.196101. 34797127

[ref45] Uhlig M. R.; Benaglia S.; Thakkar R.; Comer J.; Garcia R. Atomically Resolved Interfacial Water Structures on Crystalline Hydrophilic and Hydrophobic Surfaces. Nanoscale 2021, 13 (10), 5275–5283. 10.1039/D1NR00351H. 33624666

[ref46] Martin-Jimenez D.; Chacon E.; Tarazona P.; Garcia R. Atomically Resolved Three-Dimensional Structures of Electrolyte Aqueous Solutions near a Solid Surface. Nat. Commun. 2016, 7. 10.1038/ncomms12164. PMC4947176 27416784

[ref47] Marutschke C.; Walters D.; Cleveland J.; Hermes I.; Bechstein R.; Kühnle A. Three-Dimensional Hydration Layer Mapping on the (10.4) Surface of Calcite Using Amplitude Modulation Atomic Force Microscopy. Nanotechnology 2014, 25 (33), 335703 10.1088/0957-4484/25/33/335703. 25074402

[ref48] Nellist M. R.; Laskowski F. A. L.; Qiu J.; Hajibabaei H.; Sivula K.; Hamann T. W.; Boettcher S. W. Potential-Sensing Electrochemical Atomic Force Microscopy for in Operando Analysis of Water-Splitting Catalysts and Interfaces. Nat. Energy 2018, 3 (1), 46–52. 10.1038/s41560-017-0048-1.

[ref49] Hirata K.; Kitagawa T.; Miyazawa K.; Okamoto T.; Fukunaga A.; Takatoh C.; Fukuma T. Visualizing Charges Accumulated in an Electric Double Layer by Three-Dimensional Open-Loop Electric Potential Microscopy. Nanoscale 2018, 10 (30), 14736–14746. 10.1039/C8NR03600D. 30042993

[ref50] Weaver M. J.; Gao X. In-Situ Electrochemical Surface Science. Annu. Rev. Phys. Chem. 1993, 44 (1), 459–494. 10.1146/annurev.pc.44.100193.002331.

[ref51] Giessibl F. J. Atomic Force Microscopy with qPlus Sensors. MRS Bulletin. Springer Nature May 1, 2024; pp 492–502. 10.1557/s43577-023-00654-w.

[ref52] Wutscher E.; Giessibl F. J. Atomic Force Microscopy at Ambient and Liquid Conditions with Stiff Sensors and Small Amplitudes. Rev. Sci. Instrum. 2011, 82 ( (9), ). 10.1063/1.3633950. 21974590

[ref53] Weymouth A. J.; Wastl D.; Giessibl F. J. Advances in AFM: Seeing Atoms in Ambient Conditions. In e-Journal of Surface Science and Nanotechnology; Surface Science Society of Japan, 2018; Vol. 16, pp 351–355. 10.1380/ejssnt.2018.351.

[ref54] Wastl D. S.; Weymouth A. J.; Giessibl F. J. Optimizing Atomic Resolution of Force Microscopy in Ambient Conditions. Phys. Rev. B Condens Matter Mater. Phys. 2013, 87 ( (24), ). 10.1103/PhysRevB.87.245415.

[ref55] Wastl D. S.; Judmann M.; Weymouth A. J.; Giessibl F. J. Atomic Resolution of Calcium and Oxygen Sublattices of Calcite in Ambient Conditions by Atomic Force Microscopy Using qPlus Sensors with Sapphire Tips. ACS Nano 2015, 9 (4), 3858–3865. 10.1021/acsnano.5b01549. 25816927

[ref56] Pürckhauer K.; Maier S.; Merkel A.; Kirpal D.; Giessibl F. J. Combined Atomic Force Microscope and Scanning Tunneling Microscope with High Optical Access Achieving Atomic Resolution in Ambient Conditions. Rev. Sci. Instrum. 2020, 91 ( (8), ). 10.1063/5.0013921. 32872933

[ref57] Pürckhauer K.; Weymouth A. J.; Pfeffer K.; Kullmann L.; Mulvihill E.; Krahn M. P.; Müller D. J.; Giessibl F. J. Imaging in Biologically-Relevant Environments with AFM Using Stiff qPlus Sensors. Sci. Rep 2018, 8 ( (1), ). 10.1038/s41598-018-27608-6. PMC6008343 29921947

[ref58] Auer A.; Eder B.; Giessibl F. J. Electrochemical AFM/STM with a qPlus Sensor: A Versatile Tool to Study Solid-Liquid Interfaces. J. Chem. Phys. 2023, 159 ( (17), ). 10.1063/5.0168329. 37909458

[ref59] Giessibl F. J. Advances in Atomic Force Microscopy. Rev. Mod. Phys. 2003, 75 (3), 949–983. 10.1103/RevModPhys.75.949.

[ref60] Fukuma T. Water Distribution at Solid/Liquid Interfaces Visualized by Frequency Modulation Atomic Force Microscopy. Sci. Technol. Adv. Mater. 2010, 11 (3), 033003 10.1088/1468-6996/11/3/033003. 27877337 PMC5074298

[ref61] García R. Amplitude Modulation Atomic Force Microscopy; Wiley, 2010. 10.1002/9783527632183.

[ref62] Putman C. A. J.; Van Der Werf K. O.; De Grooth B. G.; Van Hulst N. F.; Greve J. Tapping Mode Atomic Force Microscopy in Liquid. Appl. Phys. Lett. 1994, 64 (18), 2454–2456. 10.1063/1.111597.

[ref63] Fukuma T. Water Distribution at Solid/Liquid Interfaces Visualized by Frequency Modulation Atomic Force Microscopy. Sci. Technol. Adv. Mater. 2010, 11 (3), 033003 10.1088/1468-6996/11/3/033003. 27877337 PMC5074298

[ref64] Tamayo J.; García R. Deformation, Contact Time, and Phase Contrast in Tapping Mode Scanning Force Microscopy. Langmuir 1996, 12 (18), 4430–4435. 10.1021/la960189l.

[ref65] Anczykowski B.; Krüger D.; Fuchs H. Cantilever Dynamics in Quasinoncontact Force Microscopy: Spectroscopic Aspects. Phys. Rev. B 1996, 53 (23), 15485–15488. 10.1103/PhysRevB.53.15485. 9983377

[ref66] Umeda K.; Kobayashi K.; Minato T.; Yamada H. Atomic-Level Viscosity Distribution in the Hydration Layer. Phys. Rev. Lett. 2019, 122 ( (11), ). 10.1103/PhysRevLett.122.116001. 30951327

[ref67] Minato T.; Umeda K.; Kobayashi K.; Araki Y.; Konishi H.; Ogumi Z.; Abe T.; Onishi H.; Yamada H. Atomic-Level Nature of Solid/Liquid Interface for Energy Conversion Revealed by Frequency Modulation Atomic Force Microscopy. Jpn. J. Appl. Phys.. IOP Publishing Ltd September 1, 2021. 10.35848/1347-4065/abffa2.

[ref68] Hembacher S.; Giessibl F. J.; Mannhart J.; Quate C. F. Revealing the Hidden Atom in Graphite by Low-Temperature Atomic Force Microscopy. Proc. Natl. Acad. Sci. U. S. A. 2003, 100 (22), 12539–12542. 10.1073/pnas.2134173100. 14504395 PMC240651

[ref69] Liang Y.; Pfisterer J. H. K.; McLaughlin D.; Csoklich C.; Seidl L.; Bandarenka A. S.; Schneider O. Electrochemical Scanning Probe Microscopies in Electrocatalysis. Small Methods. John Wiley and Sons Inc. August 1, 2019. 10.1002/smtd.201800387.

[ref70] Liebig A.; Setescak C.; Weindl A.; Giessibl F. J. Structural Characterization of Defects in the Topological Insulator Bi2Se3 at the Picometer Scale. J. Phys. Chem. C 2022, 126 (51), 21716–21722. 10.1021/acs.jpcc.2c06084.

[ref71] Fang Y.; Ding S. Y.; Zhang M.; Steinmann S. N.; Hu R.; Mao B. W.; Feliu J. M.; Tian Z. Q. Revisiting the Atomistic Structures at the Interface of Au(111) Electrode-Sulfuric Acid Solution. J. Am. Chem. Soc. 2020, 142 (20), 9439–9446. 10.1021/jacs.0c02639. 32338907

[ref72] Schneeweiss M. A.; Kolb D. M.; Liu D.; Mandler D. Anodic Oxidation of Au(111). Can. J. Chem. 1997, 75 (11), 1703–1709. 10.1139/v97-603.

[ref73] Gross L.; Mohn F.; Moll N.; Liljeroth P.; Meyer G. The Chemical Structure of a Molecule Resolved by Atomic Force Microscopy. Science (1979) 2009, 325 (5944), 1110–1114. 10.1126/science.1176210. 19713523

[ref74] Mönig H.; Hermoso D. R.; Arado O. D.; Todorović M.; Timmer A.; Schüer S.; Langewisch G.; Pérez R.; Fuchs H. Submolecular Imaging by Noncontact Atomic Force Microscopy with an Oxygen Atom Rigidly Connected to a Metallic Probe. ACS Nano 2016, 10 (1), 1201–1209. 10.1021/acsnano.5b06513. 26605698

[ref75] Voïtchovsky K. High-Resolution AFM in Liquid: What about the Tip?. Nanotechnology 2015, 26 (10), 100501 10.1088/0957-4484/26/10/100501. 25687326

[ref76] Akrami S. M. R.; Nakayachi H.; Watanabe-Nakayama T.; Asakawa H.; Fukuma T. Significant Improvements in Stability and Reproducibility of Atomic-Scale Atomic Force Microscopy in Liquid. Nanotechnology 2014, 25 (45), 455701 10.1088/0957-4484/25/45/455701. 25327221

[ref77] Miyazawa K.; Tracey J.; Reischl B.; Spijker P.; Foster A. S.; Rohl A. L.; Fukuma T. Tip Dependence of Three-Dimensional Scanning Force Microscopy Images of Calcite-Water Interfaces Investigated by Simulation and Experiments. Nanoscale 2020, 12 (24), 12856–12868. 10.1039/D0NR02043E. 32520063

[ref78] Nakouzi E.; Stack A. G.; Kerisit S.; Legg B. A.; Mundy C. J.; Schenter G. K.; Chun J.; De Yoreo J. J. Moving beyond the Solvent-Tip Approximation to Determine Site-Specific Variations of Interfacial Water Structure through 3D Force Microscopy. J. Phys. Chem. C 2021, 125 (2), 1282–1291. 10.1021/acs.jpcc.0c07901.

[ref79] Hahn J. R.; Ho W. Single Molecule Imaging and Vibrational Spectroscopy with a Chemically Modified Tip of a Scanning Tunneling Microscope. Phys. Rev. Lett. 2001, 87 ( (19), ). 10.1103/PhysRevLett.87.196102. 11690431

[ref80] Ito T.; Bühlmann P.; Umezawa Y. Scanning Tunneling Microscopy Using Chemically Modified Tips. Anal. Chem. 1998, 70 (2), 255–259. 10.1021/ac970498w.

